# Chest Radiography Use in Hospitalized Children with Acute Respiratory Tract Infections: A Baseline Analysis for Imaging Optimization

**DOI:** 10.3390/children13040505

**Published:** 2026-04-03

**Authors:** Roxana Axinte, Sorin Axinte, Elena Tătăranu, Laura Ion, Adina Mihaela Frenți, Florin Filip, Gabriela Burțilă, Liliana Anchidin-Norocel, Smaranda Diaconescu

**Affiliations:** 1 Doctoral School, George Emil Palade University of Medicine, Pharmacy, Science and Technology, 540142 Targu Mures, Romania; 2 Clinical Department of Pediatrics, Sf. Ioan cel Nou, Emergency Hospital, 720224 Suceava, Romania; 3 Faculty of Medicine and Biological Sciences, Stefan cel Mare University of Suceava, 720229 Suceava, Romania; florin.filip@usm.ro (F.F.);; 4 Department of ENT, Sf. Ioan cel Nou, Emergency Hospital, 720224 Suceava, Romania; 5 Faculty of Medicine, University Titu Maiorescu, 040441 Bucharest, Romania; laura.ion@prof.utm.ro (L.I.);; 6 Clinical Department of Neonatology, Sf. Ioan cel Nou, Emergency Hospital, 720224 Suceava, Romania; 7 Department of Pediatric Surgery, Sf. Ioan cel Nou, Emergency Hospital, 720224 Suceava, Romania; 8 Department of Pediatrics, Sf. Maria Emergency Children’s Hospital, 700309 Iasi, Romania

**Keywords:** pediatric respiratory infections, chest radiography, diagnostic stewardship, radiation exposure, quality improvement, pediatric emergency care, imaging optimization

## Abstract

**Background:** Pediatric respiratory infections represent a leading cause of emergency department (ED) visits and hospitalizations. Chest X-rays are frequently used in their diagnostic evaluation, despite guideline recommendations advocating restrictive imaging strategies, particularly in young children with uncomplicated disease. Excessive imaging raises concerns regarding cumulative radiation exposure and inefficient resource utilization. **Objectives**: To quantify potentially unnecessary chest radiography use in hospitalized pediatric patients with respiratory infections and to identify age-related and diagnostic patterns suitable for targeted imaging optimization interventions. **Methods:** We conducted a retrospective observational study analyzing pediatric patients presented to the ED of a tertiary county hospital in Romania over a period of 12 months. Data regarding respiratory diagnoses, hospitalization status, patient age, and chest radiography utilization were extracted from electronic medical records. **Results:** Among more than 26,000 pediatric emergency presentations, 4139 children required hospitalization, of whom 1212 were diagnosed with respiratory infections. A total of 3414 chest radiographs were performed, with the highest imaging burden observed in children aged 0–4 years. Repeated imaging was common in interstitial pneumonia, bronchiolitis, and bronchial hyperreactivity. A strong negative correlation was identified between patient age and imaging frequency (r = −0.70, *p* < 0.001). **Conclusions:** Thoracic radiographs are disproportionately used in young children with respiratory infections, particularly in conditions with limited imaging indications. These findings provide an essential baseline for the development of targeted quality improvement interventions aimed at reducing unnecessary pediatric imaging.

## 1. Introduction

Respiratory infections are among the leading causes of morbidity, emergency department visits, and hospital admissions in children worldwide [1,2,3]. In clinical practice, chest radiography remains a commonly used diagnostic tool for evaluating pediatric respiratory symptoms, despite growing evidence indicating that imaging is unnecessary in many uncomplicated cases, particularly those of viral etiology [4,5,6].

Children are especially vulnerable to the potential risks associated with ionizing radiation due to increased tissue radiosensitivity and longer life expectancy, which may amplify long-term stochastic effects [7,8]. Consequently, international guidelines recommend restrictive imaging strategies in pediatric respiratory infections, emphasizing clinical severity and suspected complications as the primary indications for chest radiography [9,10].

Despite these recommendations, real-world data continue to demonstrate substantial variability in imaging practices. Factors such as diagnostic uncertainty, parental expectations, and changes in clinical behavior following the COVID-19 pandemic may contribute to persistent overuse [11,12]. These observations highlight the need for institutional-level evaluations of imaging utilization to identify targets for diagnostic stewardship interventions. However, data describing real-world imaging practices in pediatric respiratory infections remain limited, particularly in Eastern European healthcare systems. Institutional analyses are therefore essential to identify patterns of potential imaging overuse and to support targeted optimization strategies [13]. The aim of this study was to quantify chest radiography use in pediatric patients hospitalized with respiratory infections and to identify clinical scenarios and age groups in which imaging optimization interventions may be most effective.

## 2. Materials and Methods

This study is a retrospective observational analysis conducted at “Sfântul Ioan cel Nou” County Clinical Hospital in Suceava, Romania, over a 12-month period. Sfântul Ioan cel Nou’ County Clinical Hospital is a tertiary-level public hospital and the main referral center for pediatric care in Suceava County and surrounding regions. The hospital provides emergency and inpatient pediatric services to a large pediatric population and includes both the Pediatric and Infectious Diseases departments. The study evaluated pediatric patients managed within the Emergency Department (ED), as well as those admitted to the Pediatric and Infectious Diseases departments (Table 1).

The study population included all pediatric patients aged 0–18 years who were evaluated or hospitalized during the study period. More than 26,000 pediatric presentations were evaluated in the Emergency Unit during the study period, of which 4139 cases required hospitalization in the Pediatric and Infectious Diseases departments. During the study period, the Pediatric Department included 89 inpatient beds, with a mean length of hospital stay of 3.6 days and an annual bed occupancy rate of approximately 60–70%, resulting in a high patient turnover that is consistent with the reported annual number of pediatric hospitalizations. Within the hospitalized cohort, 1212 patients were diagnosed with respiratory infections and included in the subsequent analysis. Respiratory symptoms at presentation were not systematically coded as a separate searchable variable in the electronic medical record system, which limited the possibility of reliably identifying all patients presenting with respiratory complaints. Accordingly, the present analysis focuses on imaging utilization among hospitalized children with confirmed respiratory infections and does not include all patients presenting to the Emergency Department with respiratory symptoms. The distribution of respiratory cases and the use of radiological investigations were assessed separately for each department, in accordance with clinical protocols, diagnostic indications, and disease severity, with imaging performed selectively based on clinical judgment. No standardized clinical severity scoring system was available in the retrospective dataset, and disease severity was assessed based on the clinical evaluation documented by the attending physician. Given the retrospective design of the study, the indication for chest radiography was determined by the attending physician according to clinical judgment and local practice patterns rather than by a standardized institutional imaging protocol.

The informed consent forms were signed by the legal guardians at the moment of the presentation in the ED.

Data were collected retrospectively from the hospital’s electronic medical records system. All personal data were anonymized to ensure participant confidentiality. Extracted variables included patient age, the type of clinical presentation, hospitalization status, primary diagnosis, and the use of radiological investigations. Respiratory infection diagnoses were established based on clinical assessment documented in the electronic medical records, including the presence of respiratory symptoms (such as cough, tachypnea, wheezing, or respiratory distress), physical examination findings, and, when available, laboratory or imaging data, according to the attending physician’s evaluation. Diagnoses were recorded according to ICD-10 classification codes.

This study was approved by the Ethics Committee of “Sfântul Ioan cel Nou” County Clinical Hospital, Suceava, Romania (approval no. 49/31.10.2024).

The statistical analysis was performed using the IBM SPSS Statistics for Windows, v20.0 (Armonk, NY, USA). Descriptive statistics included percentages, means, and standard deviations. The chi-square test and Pearson correlation were used for inferential statistical analysis. A threshold *p* < 0.05 was considered statistically significant. Schematic illustrations were generated using BioRender.com (RRID: SCR_018361).

## 3. Results

**Figure 1:**
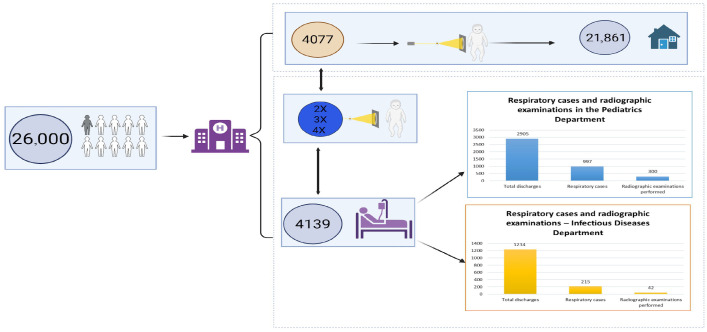
Flowchart of pediatric patient distribution and respiratory case selection.

During the study period, more than 26,000 pediatric patients were evaluated in the ED. Most cases (21,861 patients) were managed on an outpatient basis and discharged without hospitalization, while 4139 pediatric patients required inpatient admission to the Pediatric and Infectious Diseases departments (Figure 1).

Respiratory infections accounted for a substantial proportion of hospital admissions, with 1212 cases identified among hospitalized children. These patients were managed either in the Pediatric Department or the Infectious Diseases Department, depending on clinical severity and diagnostic complexity.

**Figure 2:**
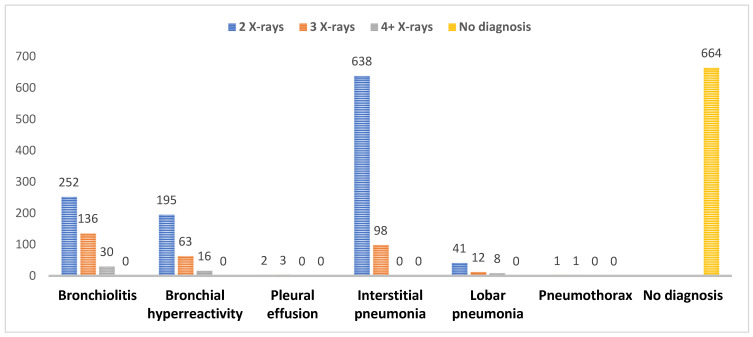
Distribution of chest radiographs by respiratory diagnosis.

Among hospitalized patients, multiple radiographic examinations were frequently performed, with some children undergoing two, three, or even four chest radiographs (Figure 2). The highest number of repeated radiographic examinations was observed in cases of interstitial pneumonia, followed by bronchiolitis and bronchial hyperresponsiveness. A total of 664 radiographic examinations were not associated with a definitive respiratory diagnosis. These radiographs were performed during the initial diagnostic evaluation of children presenting with respiratory symptoms (such as cough or dyspnea) or with nonspecific clinical presentations, such as fever without an evident infectious focus. In some cases, although imaging was obtained as part of the differential diagnostic assessment, a definitive respiratory infection diagnosis was not ultimately established.

**Figure 3:**
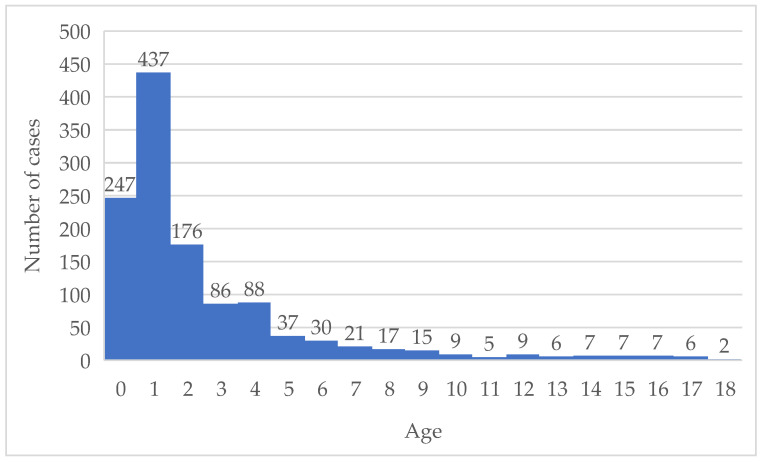
Age-related distribution of hospitalized pediatric patients.

The age distribution analysis indicated a clear predominance of cases occurring in early childhood. The highest case frequency was observed in infants and children aged 0–4 years, followed by a marked decline with increasing age. Relatively low numbers were recorded among school-aged children and adolescents. This age-related pattern was consistent across both departments (Figure 3).

Overall, 3414 chest radiographic examinations were recorded, representing imaging episodes rather than unique patients, as multiple examinations were frequently performed in the same child. The imaging burden was highest among children aged 0–4 years, who accounted for 1984 examinations, compared with 1430 examinations in the 5–15 years age group. Mean and maximum numbers of radiographs per patient were also higher in younger children. Radiographic activity varied across clinical settings, with the ED accounting for the largest share of examinations, followed by the Pediatric and Infectious Diseases departments. Minimum values remained consistently low across age groups.

The statistical analysis highlighted a strong negative correlation between patients’ age and the number of chest radiographs performed (Pearson correlation coefficient r = −0.70; *p* = 0.000803). The longitudinal analysis of the institutional records showed a progressive increase in the number of pediatric chest radiographs over time.

## 4. Discussion

This study demonstrates a substantial use of chest radiography in hospitalized children with respiratory infections, with significant rates of examinations performed in infants and young children. The high frequency of repeated radiographic examinations observed in interstitial pneumonia, bronchiolitis, and bronchial hyperresponsiveness is particularly notable, as these conditions are predominantly associated with viral etiologies and have limited indications for routine imaging according to the current guidelines [1,2,6]. In particular, current clinical guidelines indicate that even a single chest radiograph is rarely justified in non-severe bronchiolitis or bronchospasm, especially in patients who do not require advanced respiratory support such as high-flow nasal cannula (HFNC), as summarized in Table 2.

A considerable number of chest radiographs were performed in the absence of a definitive respiratory diagnosis, suggesting potential overuse of imaging in low-risk clinical scenarios. Similar patterns have been reported in pediatric emergency settings, where radiographic findings often do not alter clinical management but may contribute to increased antibiotic prescribing and prolonged hospital stays [32,33].

As expected, imaging frequency was higher in younger children, which is consistent with previous studies reporting increased radiographic utilization in infants and preschool-aged patients. The strong negative correlation between patient age and the number of radiographic examinations confirms that younger children are disproportionately exposed to diagnostic radiation. This finding is consistent with previous studies reporting higher imaging rates in infants and preschool children presenting with respiratory symptoms [7,12]. Given the increased radiosensitivity of developing tissues and the cumulative nature of radiation exposure, this age-related imbalance raises important safety concerns [34].

The observed post-pandemic increase in pediatric chest radiography mirrors international reports describing heightened diagnostic vigilance and a lower threshold for imaging in respiratory illnesses following the COVID-19 pandemic [6]. This trend may be explained by several factors, including increased awareness of respiratory complications, evolving clinical protocols, diagnostic uncertainty in young children, parental expectations, and institutional practice patterns. In addition, the absence of standardized clinical decision-support tools for imaging may have further lowered the threshold for ordering chest radiographs in selected clinical scenarios. The variability in clinical decision-making among European pediatricians has been previously documented, emphasizing the influence of local practice patterns, institutional culture, and clinician perception on diagnostic and therapeutic choices [35].

Previous studies have demonstrated that repeated medical interventions and investigations may negatively influence pediatric quality of life, particularly in vulnerable populations [36].

Taken together, these findings underscore the need for targeted diagnostic stewardship strategies aimed at optimizing imaging use in pediatric respiratory infections. The issue of medical overuse extends beyond diagnostic imaging. Previous pediatric studies from Romania have highlighted the consequences of excessive therapeutic interventions, including increased antimicrobial resistance, reinforcing the importance of data-driven strategies to optimize clinical decision-making across pediatric specialties [37]. Interventions focusing on younger age groups and conditions with predominantly viral etiologies may offer the greatest potential for reducing unnecessary radiation exposure while maintaining diagnostic safety. International guidelines emphasize clinical assessment over routine imaging in bronchiolitis and viral pneumonia, supporting targeted interventions to reduce unnecessary chest radiography [9,10]. Quality improvement initiatives combining clinical pathways, physician education, and decision-support tools have been shown to safely reduce imaging rates without adverse clinical outcomes [6,12].

Beyond post-pandemic clinical vigilance, several institutional and systemic factors may contribute to increased imaging utilization. Defensive medicine practices, particularly in emergency settings characterized by diagnostic uncertainty, may lower the threshold for ordering radiologic examinations. In pediatric care, where symptoms are often nonspecific and clinical assessment may be challenging, physicians may rely more readily on imaging to exclude serious pathology and mitigate perceived medicolegal risk [38,39,40].

Previous studies have shown that a substantial proportion of radiological examinations are not consistently justified in routine clinical practice. Rawle and Pighills reported that nearly half of emergency department radiographic examinations were considered unjustified, while international analyses have estimated that between 30% and 77% of high-dose imaging procedures may lack adequate justification [41]. These findings persist despite existing regulatory frameworks intended to promote appropriate referral practices.

In this context, the absence of a structured radiation stewardship framework, including systematic dose monitoring, cumulative exposure tracking, and institutional imaging appropriateness oversight, may represent an important gap contributing to variability in imaging practices [42,43].

Institutional records indicate that 2128 chest radiographs were performed in 2018, increasing to 2648 in 2019, and reaching 4077 examinations in 2024. However, these figures represent absolute counts only. The observed increase in the absolute number of chest radiographs over previous years should be interpreted cautiously, as corresponding denominator data (e.g., total hospitalizations or emergency department visits) were not available for those periods. Therefore, no formal temporal comparisons were performed.

This study has several limitations that should be acknowledged. Firstly, this is a single-center observational study limited to a 12-month period, which may limit the generalizability of the findings. Secondly, detailed clinical information was not consistently available, limiting the ability to correlate radiographic findings with clinical presentation and disease severity.

Another limitation of this study is that the decision to perform chest radiography relied on the clinical judgment of the attending physician rather than on a standardized institutional protocol. As a result, some variability in imaging practices between clinicians may have occurred, reflecting routine clinical decision-making in a real-world pediatric emergency setting. Future research should explore whether the introduction of standardized clinical decision-support tools could help reduce unnecessary imaging in pediatric respiratory infections.

In addition, the impact of radiographic findings on clinical decision-making (e.g., initiation of antibiotics or changes in treatment strategy) could not be systematically evaluated, as this information was not consistently recorded in the electronic medical records used for this retrospective analysis. This limitation restricted our ability to assess how imaging results influenced patient management. Furthermore, standardized clinical severity scores were not consistently documented in the electronic medical records, which limited our ability to stratify patients according to objective severity criteria. Finally, due to limitations in the structure of the retrospective dataset, it was not possible to determine whether repeated radiographs were performed within the same department or across different hospital departments.

## 5. Conclusions

In conclusion, this study demonstrates a substantial and increasing use of chest radiography among pediatric patients evaluated in the ED, with the greatest imaging burden observed in infants and young children. Respiratory infections represented a major driver of hospital admissions and radiographic examinations, and repeated imaging was frequently performed, particularly in children with interstitial pneumonia and bronchiolitis. Given the higher radiosensitivity of pediatric patients and their longer life expectancy, even low-dose exposures may carry a cumulative risk. Careful clinical assessment and strict adherence to evidence-based imaging guidelines should therefore be prioritised. Optimising the appropriateness of imaging requests and avoiding unjustified or repeated examinations represent key strategies to improving patient safety while maintaining high standards of diagnostic care. These findings support the implementation of institutional imaging stewardship programs aimed at reducing unnecessary pediatric chest radiography while maintaining diagnostic safety.

## Figures and Tables

**Table 1:** Inclusion and exclusion criteria.

Inclusion Criteria	Exclusion Criteria
Children aged 0–18 years	Patients older than 18
Presentation to the Emergency Department followed by hospitalization	Elective hospitalizations
Thoracic X-ray	No thoracic X-ray

**Table 2:** Etiologies of pediatric respiratory tract infections and highlights clinical scenarios in which routine chest radiography has limited diagnostic value.

Clinical Syndrome	Predominant Etiologies	Typical Age Group	Role of Chest Radiography	Remarks Relevant to Imaging Optimization	Local Practice Patterns
Upper respiratory tract infection (rhinitis, nasopharyngitis, pharyngitis) [14]	Viral: rhinovirus, seasonal coronaviruses, adenovirus, influenza, parainfluenza	All pediatric ages	Not indicated	Diagnosis is clinical; chest radiography does not alter management in uncomplicated cases.	Radiography occasionally performed in febrile children with persistent cough or nonspecific respiratory symptoms.
Acute bronchitis [15,16]	Viral: rhinovirus, influenza, respiratory syncytial virus (RSV); Rare bacterial: *Bordetella pertussis*	Preschool and school-aged children	Not routinely indicated	Imaging reserved for atypical presentation or suspicion of pneumonia.	Radiography occasionally performed to exclude pneumonia in cases with equivocal clinical findings or prolonged symptoms.
Bronchiolitis [17,18,19,20]	Viral: RSV, rhinovirus, human metapneumovirus, parainfluenza	Infants <2 years	Not recommended routinely	Radiographic findings are often nonspecific and may lead to unnecessary antibiotic use.	Radiography is frequently performed in ED due to diagnostic uncertainty, especially in febrile infants or those presenting with moderate respiratory distress.
Viral-induced wheezing/bronchial hyperreactivity [21,22]	Viral: rhinovirus, RSV	Infants and young children	Not routinely indicated	High rates of imaging despite limited diagnostic yield in uncomplicated cases.	Radiography commonly performed during first wheezing episodes to exclude pneumonia.
Interstitial pneumonia (suspected viral pneumonia) [23,24]	Viral: influenza, RSV, metapneumovirus, adenovirus	Young children	Selective use only	Routine imaging rarely changes management in clinically stable patients.	Radiography often performed when clinical findings are equivocal.
Community-acquired pneumonia, non-severe [25,26]	Bacterial: *Streptococcus pneumoniae*, *Haemophilus influenzae*; Atypical: *Mycoplasma pneumoniae*	All ages	Conditional	Imaging may be considered in moderate–severe disease or diagnostic uncertainty.	Radiography commonly obtained when presentation is unclear or symptoms persist.
Post-viral bacterial pneumonia [27,28]	*Staphylococcus aureus*, *Streptococcus pneumoniae*	School-aged children	Indicated	Imaging appropriate in clinical deterioration or suspected complications.	Radiography routinely performed
Complicated pneumonia (effusion, empyema) [29,30,31]	Bacterial	All ages	Indicated	Radiography and further imaging essential for management decisions.	Radiography performed systematically to guide therapeutic interventions.

## Data Availability

The original contributions presented in this study are included in the article. Further inquiries can be directed to the corresponding authors.
